# Inhibitory Potential of Chromene Derivatives on Structural and Non-Structural Proteins of Dengue Virus

**DOI:** 10.3390/v14122656

**Published:** 2022-11-28

**Authors:** Babitha Thekkiniyedath Dharmapalan, Raja Biswas, Sathianarayanan Sankaran, Baskar Venkidasamy, Muthu Thiruvengadam, Ginson George, Maksim Rebezov, Gokhan Zengin, Monica Gallo, Domenico Montesano, Daniele Naviglio, Mohammad Ali Shariati

**Affiliations:** 1 Department of Pharmaceutical Chemistry, Amrita School of Pharmacy, AIMS Health Sciences Campus, Amrita Vishwa Vidyapeetham, Kochi 682041, India; 2 Centre for Nanosciences and Molecular Medicine, Amrita Vishwa Vidyapeetham, Kochi 682041, India; 3 Faculty of Pharmacy, Karpagam Academy of Higher Education, Karpagam University, Pollachi Main Road, Eachanari Post, Coimbatore 641021, India; 4 Department of Oral and Maxillofacial Surgery, Saveetha Dental College and Hospitals, Saveetha Institute of Medical and Technical Sciences, Saveetha University, Chennai 600077, India; 5 Department of Applied Bioscience, College of Life and Environmental Sciences, Konkuk University, Seoul 05029, Republic of Korea; 6 Department of Scientific Research, K.G. Razumovsky Moscow State University of Technologies and Management (The First Cossack University), 73 Zemlyanoy Val, 109004 Moscow, Russia; 7 Department of Scientific Research, Russian State Agrarian University—Moscow Timiryazev Agricultural Academy, 49 Timiryazevskaya Str., 127550 Moscow, Russia; 8 Faculty of Biotechnology and Food Engineering, Ural State Agricultural University, 42 Karl Liebknecht Str., 620075 Yekaterinburg, Russia; 9 Department of Biology, Science Faculty, Selcuk University, Konya 42130, Turkey; 10 Department of Molecular Medicine and Medical Biotechnology, University of Naples Federico II, Via Pansini 5, 80131 Naples, Italy; 11 Department of Pharmacy, University of Naples Federico II, Via D. Montesano 49, 80131 Naples, Italy; 12 Department of Chemical Sciences, University of Naples Federico II, Via Cintia 4, 80126 Naples, Italy

**Keywords:** dengue virus, structural proteins, non-structural proteins, flavonoids, chromene, virus inhibitory potential

## Abstract

Dengue fever is a mosquito-borne viral disease that has become a serious health issue across the globe. It is caused by a virus of the Flaviviridae family, and it comprises five different serotypes (DENV-1 to DENV-5). As there is no specific medicine or effective vaccine for controlling dengue fever, there is an urgent need to develop potential inhibitors against it. Traditionally, various natural products have been used to manage dengue fever and its co-morbid conditions. A detailed analysis of these plants revealed the presence of various chromene derivatives as the major phytochemicals. Inspired by these observations, authors have critically analyzed the anti-dengue virus potential of various 4*H* chromene derivatives. Further, in silico, in vitro, and in vivo reports of these scaffolds against the dengue virus are detailed in the present manuscript. These analogues exerted their activity by interfering with various stages of viral entry, assembly, and replications. Moreover, these analogues mainly target envelope protein, NS2B-NS3 protease, and NS5 RNA-dependent RNA polymerase, etc. Overall, chromene-containing analogues exerted a potent activity against the dengue virus and the present review will be helpful for the further exploration of these scaffolds for the development of novel antiviral drug candidates.

## 1. Introduction

Dengue fever (DF) is a serious health issue worldwide that affects approximately 3.9 billion people [1] and 100–300 million people are infected every year. The spread of the dengue virus has been attributed to urbanization, climate change, population growth, and unscientific vector control, etc. [2]. According to the World Health Organization (WHO), before 1970, only nine countries were affected by DF, but in 2020, almost 130 countries were at risk [3]. Tropical and subtropical countries are primarily affected, where Asia accounts for 70% of the cases [1]. DF is spreading worldwide at an alarming rate [2], causing severe health emergencies across the globe. The dengue virus (DENV) pathogen is the causative agent of DF that belongs to the arbovirus (arthropod-borne virus) family Flaviviridae and the genus Flavivirus. It is a positive-stranded single RNA virus. Four other major types of Flavivirus are reported to date, which include West Nile, Japanese encephalitis, yellow fever, and Tick-borne encephalitis virus. Since DF is associated with major symptoms of myalgia and arthralgia, it is also called “break-bone fever” [4].

Aedes mosquitoes primarily transmit the dengue virus infection. The dengue virus first emerged in subhuman primates 500–1000 years ago and spread to humans in Africa and Southeast Asia [5]. The DENV is primarily responsible for subclinical infections, such as mild flu, but it can sometimes cause severe dengue conditions (Dengue Hemorrhagic Fever (DHF) and Dengue Shock Syndrome (DSS)). As there is no distinct medicine for dengue, symptomatic and supportive care with supplementation of fluids is given [6].

The five distinct and closely related specific dengue virus serotypes are DENV-1, DENV-2, DENV-3, DENV-4, and DENV-5. Ren Kimura and Susumu Hoffa first isolated the dengue viruses (DENV-1 and DENV-2) during dengue epidemics in Nagasaki, Japan, in 1943, and DENV-3 and DENV-4 were isolated from dengue-infected patients in the Philippines and Thailand in 1953 [7]. The newest serotype, DENV-5, was isolated from Malaysia in 2007. In humans, the dengue virus causes two types of infections: primary (DF) and secondary (DHF and DSS). DENV-1 and DENV-3 caused DHF in primary dengue infections, while DENV-2 and DENV-4 caused DHF in secondary infections [1]. The symptoms of DF include a fever of 40 °C, headache, pain, leucopenia, myalgia, arthralgia, nausea, vomiting, retro-orbital pain, rashes, and swollen glands, etc. [8,9]. Patients with DHF may experience respiratory distress, plasma leakage, fluid accumulation, internal bleeding, and organ impairment [5,10]. In contrast, severe bleeding and shock occur in DSS patients, leading to death if not treated properly [9]. People who have recovered from one dengue serotype only have immunity against that particular serotype. Secondary infection can occur if a patient has previously been affected by any of the other four serotypes [1].

## 2. Structure and Biology of Dengue Virus

**Figure 1:**
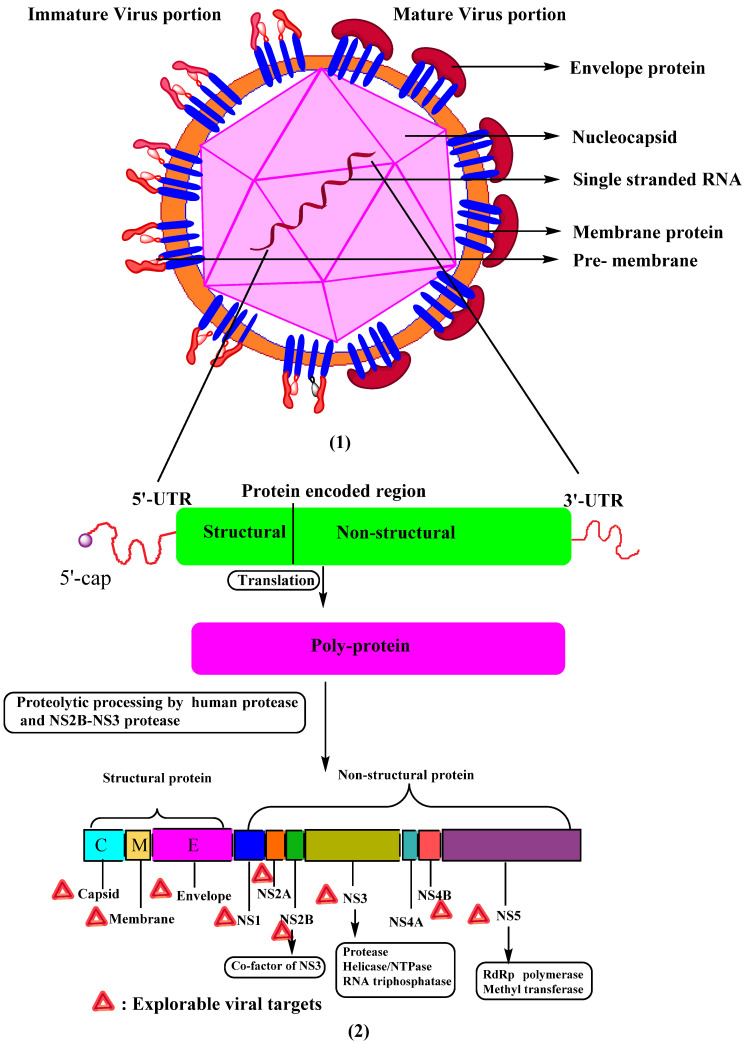
Schematic diagram showing (**1**) structure of dengue virus, mature, and immature viral portion difference. (**2**) DENV RNA genome and polyprotein processing to form three structural and seven non-structural proteins. UTR—untranslated region.

Kuhn, R.J. et al. [10] extensively studied the morphology of the DENV. They found that the virus’s surface was even and possessed an icosahedral shape (Figure 1). The virus’s external surface is coated with an envelope glycoprotein dimer, followed by a nucleocapsid that contains a viral genome linked to many copies of C-protein. The single-stranded RNA genome contains around 10,700 nucleotides with a type-1 cap at the 50 ends and lacking a 30 poly’A’ tail [8]. The genome encodes three structural proteins: an envelope (E, 495 amino acids), pre-membrane/membrane (PrM/M, 75 amino acids), and capsid (C, 100 amino acids), as well as seven non-structural (NS) proteins: NS1, NS2A, NS2B, NS3, NS4A, NS4B, and NS5 [11,12] (Figure 1). Structural proteins are present in the N′ terminus and non-structural proteins in the C′ terminus of viral polyprotein. The mature DENV virion has a smooth surface with a diameter of 50 nm, whereas the immature virion has a spiky surface with a diameter of 60 nm [11,12]. Non-structural proteins are responsible for the viral replication process, whereas structural proteins play a role in developing the DENV virion’s component.

## 3. Pathogenesis

**Figure 2:**
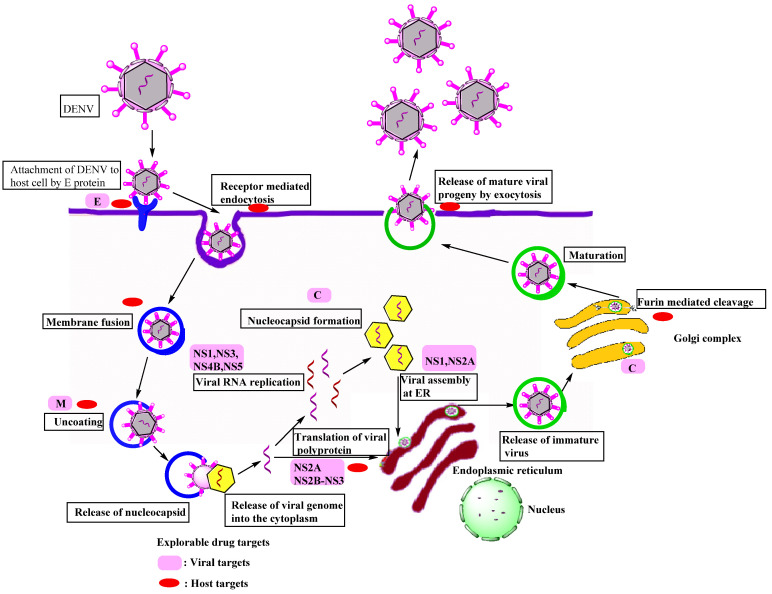
Schematic diagrams showing the life cycle of the DENV in a host cell.

DENV primarily focuses on dendritic cells (DCs), monocytes/macrophages, B cells, T cells, endothelial cells, hepatocytes, and brain cells of the host. DENV reaches the target cell in a serotype-specific manner via non-specific receptor-mediated endocytosis. The DENV life cycle is depicted in Figure 2. Heparan sulfate, DC-SIGN, CD-14, HSP-90, HSP-70, mannose receptor, GRP-78, high-affinity laminin receptor, TIM-1, TAM, AXL, and claudin-1 are some of the most well-known receptors for endocytosis [12,13,14]. Envelope proteins on virus surfaces attach to these receptors on the host cell to drive viral particles to the endocytic route. The envelope protein experiences a significant structural change in the endosome due to the lower pH. Because of these modifications, the E protein adheres to the endosomal membrane that allows the nucleocapsid to be uncoated; finally, the discharge of the viral genome to the cytoplasm occurs [14].

The discharged viral RNA undergoes two processes in the cell: translation and viral genome replication. The viral RNA functions similarly to the host mRNA. The lack of a poly-A tail in viral RNA is the most noticeable distinction from host mRNA. The translation of cellular mRNA differs from that of viral mRNA. As a result, the genome is processed into a polypeptide chain in the ribosome present on ER, where translation occurs [11]. The polypeptide chain is broken down into three structural and seven non-structural proteins by viral serine protease and cellular proteases. The host cell goes through various changes during the transformation process. These modifications forced the host cell to promote viral RNA multiplication. The emergence of a replication complex (RC), a membrane-bound microenvironment, is one of these cell alterations. Viral RNA amplification and morphogenesis are observed in RC [13]. The proteins produced by the tiny dengue viral genome perform several functions in genome replication, viral assembly, discharge of fully grown virions, and immunopathogenesis [2].

There are two phases in the viral genome replication process. In the first stage, viral positive-polarity RNA is transformed into negative-polarity RNA. These negative RNA strands are then utilized to amplify positive-strand production in the second stage. Some of the generated positive strands may become active in the translation process. The C-protein arranges the newly developed positively polar RNA strand on the ER membrane, causing a nucleocapsid to form around the genome [13]. In viral assembly, the nucleocapsid bud reaches the ER and gains access to the E and PrM proteins. The vesicular packets containing the assembled immature viral particles are moved to the Golgi apparatus, where glycosylation occurs, and the cellular endoproteases split the PrM into M-protein [12,13,15]. Various structural modifications arise during the maturation process, such as the transition from spiky immature virus particles to smooth-surfaced icosahedral mature viral particles. The infection was spread to other uninfected cells by mature viral particles that were generated from the infected cell [16,17].

## 4. Explorable Drug Targets

### 4.1. Viral Targets

Based on the pathogenesis involved in DF, numerous targets have been attracted as drug targets. The main focus of novel anti-dengue virus drug development is to prevent viral entry, RNA replication, and polyprotein cleavage. The most promising strategy for rational drug development is inhibiting viral enzymes [18]. It has the benefit of having a low toxicity and adverse effect profile; however, drug resistance develops due to fast mutation. The host enzymes have a wider spectrum of inhibitor activity and there is no risk of resistance, but there is a higher risk of cell toxicity and side effects. Structural proteins are essential for virus attachment (E protein), secretion (PrM and E protein), assembly (C protein), and non-structural proteins for enzymatic activity (NS2B3 and NS5) and regulating host immunological reactions (NS2A, NS2B3, NS4A, NS4B, and NS5); thus, these are considered as promising targets for the development of anti-dengue virus agents.

#### 4.1.1. Structural Proteins

##### Envelope Protein (E)

The virus enters the target cell through the viral envelope glycoprotein by clathrin-mediated endocytosis. It possesses 493–495 amino acids with a molecular mass of 53 kilos Dalton (kDa). The lower pH of the endosome causes the homodimers to dissociate, and their entry into the cell forms a cross-bridge between the virus and the host [19,20]. The primary purpose of the E protein is to bind and fuse with the host cell membrane. The E protein inhibitors restrict DENV from attacking in the early stages of infection. The lack of a putative active site compatible with viral proteases and polymerases is a significant obstacle in developing antivirals that target envelope proteins [21]. Each monomer comprises different domains, such as the C-terminal transmembrane anchor domain, soluble ectodomain, and stem domain, with a link between them. The hydrophobic pocket n-octyl-*β*-d-glucoside (*β*-OG) and the receptor-binding domain III are also present in each monomer. The E protein undergoes structural modifications during infection, switching from a dimeric to a trimeric orientation. These structural changes enable the DENV envelope to merge with the host endosomal membrane, releasing RNA into the cytoplasm. Thus, the envelope protein has gained significant attention in vaccine development [22].

Different DENV entry inhibitor targets are:

- **Fusion inhibitors:** Peptides and small molecules bound to the *β*-OG pocket. The peptidic inhibitors have poor absorption from the gastrointestinal tract; thus, intravenous administration is required.
- **Glycosidase inhibitors:** α-glycosidase inhibitors mainly target the glycosylation process. They are only employed in limited conditions due to their severe toxicity and lack of selectivity. Some examples are deoxynojirimycin (DNJ) and castanospermine (CSP).
- **Carbohydrate-binding agents:** These agents block the connection between the DENV envelope N-glycans and the host cell. Some examples include concanavalin A and wheat germ agglutinin.
- **Heparan mimetics:** Heparan sulfate is a receptor for DENV. Heparan mimetics contain negatively charged sulfate groups, and their steric hindrance leads to antiviral activity. Pentosan polysulfate, suramin, and fucoidan are some examples of these kinds of inhibitors [17,23].

##### PrM/Membrane Protein (M)

The PrM/M protein is made up of 175 amino acids and consists of an N-terminal region, an M domain, a stem region, and two transmembrane helices. Furin, a cellular protease, cleaves the prM protein, liberating 91 amino acids at the N-terminus but preserving 180 copies of the 75-residue core protein [17]. The M protein has a significant function in the organization and maturation of DENV [12,24]. In the Golgi apparatus, breaking occurs at the interface site between the N and the M domain, resulting in the cleavage of PrM to M-protein and eventually resulting in the maturation of the virus. It entails the reorganization of the PrM and E proteins [25].

##### Capsid Protein (C)

It is a homodimeric protein containing 100 amino acids, four α-helical sections, a disorganized N-terminal domain, and has 12 kDa molecular mass [12,15]. The C protein has a hydrophobic membrane-bridging region of 20 amino acids essential for binding to the ER. The binding of the positively charged N-terminal domain with negatively charged lipid droplets takes part in viral particle formation [11]. The C protein is essential for the development of nucleocapsids in the initial steps of the dengue virion assembly. It is also essential for prM maturation and it is a preferred target for vaccines and antivirals because it does not induce an adverse drug-enhancing response (ADE) [8,17].

##### Non-Structural Protein

Seven non-structural proteins, NS1, NS2A, NS2B, NS3, NS4A, NS4B, and NS5, are present in the C′ terminus of the viral polyprotein and mainly responsible for the viral replication process. Structural features and their functions are represented in Table 1.

### 4.2. Host Targets

Because viruses have a short genome structure, they rely on various host mechanisms to replicate. Hence, host targets serve as antiviral sites. The best-studied host proteases are furin and signal peptidase. Other targets include glucosidase, host kinases, cholesterol biosynthesis, and DHF/DSS pathogenesis, etc. Although the host targets reduced the chance for the formation of drug resistance, the toxicity to host cells remains a major concern [36]. All available literature on DENV–human interactions is accessible in the DenHunt database (http://proline.biochem.iisc.ernet.in/DenHunt/, accessed on 23 November 2022). Among these, 682 direct binding of dengue viral active sites with host proteins, 382 indirect binding, and 4120 interactions of uniquely expressed human genes in infected cell lines with affected people were reported [16].

## 5. Drugs under Clinical Trials

Currently, numerous drugs are in various stages of clinical trials and the data are summarized in Table 2.

## 6. Vector Control

Another method of reducing dengue fever is vector control; however, this negatively influences the ecosystem. Modern effective vector control systems have been developed in recent years, utilizing new biocontrol tactics, such as sterile insect techniques and the creation of genetically modified vectors [12].

## 7. Treatment

The preferred approach in anti-dengue virus treatment targets viral proteins rather than host proteins. The important requirement of DENV drug molecules is minimal toxicity, tolerable for infants, pregnant women, children, adults, and patients with other health problems; fast-acting, rapidly reducing symptoms, decreasing the incidence of disease severity; reduced interaction with other medications; and pan-serotype effect, etc. It should also have a long half-life, better solubility, stability in the gastrointestinal tract and liver, and good systemic permeability [16]. Clinical trials for potential small-molecule dengue inhibitors are underway at various stages. In either of these trials, there was no notable reduction in viremia between the drug-treated and control groups [37]. The four types of DENV (DENV1-4) were effectively inhibited in vitro by celgosivir, an α-glucosidase inhibitor, but it failed to show viral load reduction and therapeutic benefit in a Phase I b clinical trial. Two randomized, double-blind investigations of dengue patients treated with chloroquine found no therapeutic effect. In a double-blind, placebo-controlled experiment, balapiravir, an NS5 RNA-dependent RNA polymerase (RdRp) inhibitor, failed to lower viremia or fever clearance time. In preclinical toxicology investigations in mice, NITD008, a nucleoside analogue, showed substantial adverse effects. Another compound, S 610, a helicase inhibitor, has a low oral bioavailability and must be administered intravenously. Although a number of promising protease inhibitors, such as ARDP006, compound 7n, thyrothricin, etc., have been identified, their research is still in its early stages and no medications have been tested in humans so far.

In DENV patients, there is a sudden reduction in viremia [37], so patients at the earliest stage of infection should be selected for clinical trials. It is challenging to develop similarly active antibodies against all serotypes. There is currently no specific and reliable animal model that accurately replicates human DENV pathogenesis and impending progress toward a safe and effective treatment [12,38].

As there is no specific treatment for dengue fever, antivirals that are effective against DENV are the need of the hour. Significant research is ongoing in this direction, wherein the exploration of natural derivatives is one amongst them. In tropical and subtropical areas, herbal medicines are utilized by a larger number of people to treat various infections. In some countries, steam distillate of aerial parts of *Houttuynia cordata* was used for HSV 1 and influenza virus [39]. A traditional Chinese medicinal herb, *Isatis tinctoria*, is used to treat dengue virus infection [40]. In the Philippines, *Carica papaya* leaf juice was given to DF patients to improve platelet count [41]. These plants mainly comprise chromene molecules (flavonoids).

Chromene is a class of heterocyclic scaffold, widely used to design novel drug molecules [42]. Its simple structure and lesser adverse effects are beneficial for new drug development [43]. Chromene derivatives have been reported to possess a wide range of biological applications, such as anti-cancer, antimicrobial, antiviral, anti-inflammatory, anticholinesterase, monoamine oxidase (MAO) inhibition, etc. [42]. Many phytochemicals containing the chromene nucleus have potent antiviral activity against DNA and RNA viruses [44]. In this review, the authors have compiled, segregated, and analyzed various 4*H*-chromene derivatives that have been shown to have anti-DENV activities in silico, in vitro, and in vivo studies. Both natural and synthetic flavonoids contain 4*H*-chromene as their basic nucleus.

## 8. Chromene Derivatives against Dengue Virus

**Figure 3:**
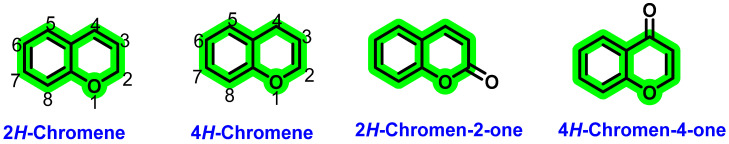
Structure of different chromene scaffolds.

The chromene or benzopyran is a heterocyclic system comprising a phenyl ring fused with a pyran ring [45]. Amongst the carbons present in chromene, eight carbons are sp^2^ hybridized, while one carbon is sp^3^ hybridized in nature. Based on the arrangement of sp^3^ hybridized carbons, chromenes can be further differentiated into 2*H*-chromene and 4*H*-chromene [45] (Figure 3). Various natural product-derived phytochemicals obtained from chromene were reported to possess different pharmacological activities. These include antioxidants [46], antimicrobial*s* [47], anticancer [46], antiviral (Kaempferol, *Rhodiola rosea* roots against H1N1 [48]), anti-fungal [49], anti-obesity [50], anti-inflammatory [51], Alzheimer’s disease [52,53], cardiovascular diseases (Quercetin, naringenin [54]), and coronary artery disease [55,56]. Antiviral activities of various chromene derivatives were also previously explored. For instance, gramniphenols (*2H*-chromene) isolated from *Arundina gramnifolia* have been extensively studied by researchers for their anti-tobacco mosaic virus activity, showing IC_50_ values 20.8, 40.8, and 57.7 Μμ [57] in different research. The overall antiviral activity of chromene relies on various factors, such as attachment of other pharmacophoric features as well as their attachment position, presence of double bond (C2–C3) in the C ring, etc. [43]. Although a few reports are available on the antiviral activities of chromene scaffolds, a detailed compilation of various pharmacophoric features is not available. To shed light on these lacunae, in the present manuscript, the authors reviewed various chromene-derived natural products for their antiviral activities along with their pharmacological activities. By considering the advantages of the chromene scaffold, the present manuscript will be helpful for the exploration of these scaffolds for the further development of novel antiviral agents. 4*H*-chromene derivatives have been utilized extensively for decades. We mainly focused on the 4*H*-chromene derivatives. 4*H*-chromene contains a benzene ring fused with a 4*H*-pyran ring at the 5,6-position. The different activities of 4*H*-chromene are mainly due to the folded nature along the oxygen axis [43]. The different antiviral effects of chromene derivatives arise from the attachment of various substituents.

Natural and synthetic flavonoids belong to this class of chromene derivatives. The basic skeleton of flavonoids is C_6_-C_3_-C_6_. The different categories of flavonoids arise because of the different oxidation levels of the three-carbon units in the C ring. Flavonoids have various subclasses: flavones, flavonol, flavanol, flavanolol, flavanones, isoflavones, and anthocyanidins [44,58]. Based on the ability to inhibit a particular target, the authors reviewed the potential of various chromene analogues, which are summarized in the later parts.

### 8.1. Chromene Analogues Acting on NS-1

**Figure 4:**
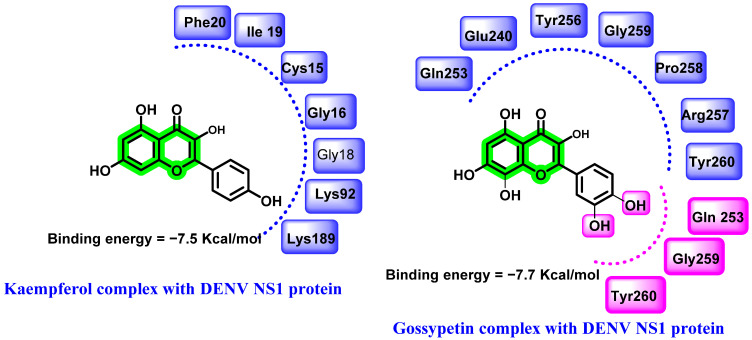
Hydrogen bonding (pink) and hydrophobic interactions (blue) of kaempferoland gossypetin with DENV NS1protein.

In a study conducted by Keramagi et al., 107 natural compounds derived from 43 medicinal plants were screened *(computational)* for their DENV and CHIKV inhibitory properties. Amongst the screened analogues, kaempferol and chymopain (isolated from *Carica papaya*) and gossypetin (isolated from *Hibiscus sabdariffa*) were found to be the lead compounds, with a binding energy of −7.5, −5.9, and −7.7 Kcal/mol, respectively [59,60] (Figure 4). Molecular dynamic study further supported the stability of these analogues in a dynamic environment. These natural compounds were also screened for their drug-likeness prediction (Lipinski’s rule of five) and ADMET profile prediction. The lead molecules chosen exhibited optimal drug likeliness and ADMET characteristics.

### 8.2. Chromene Analogues Acting on NS2B-NS3 Protease

**Figure 5:**
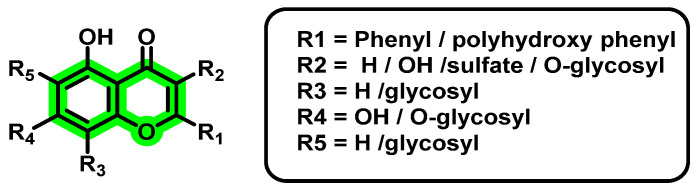
Common scaffold generated from Structure Activity Relationship study.

Sarwar et al. [61] evaluated the role of plant flavonoids against DENV NS2B-NS3 protein and, based on the obtained results, SAR and QSAR models were developed. Thus, 2FOM has been used as protein targets, and a catalytic triad of His51, Asp75, and Ser135 was used for the prediction of binding affinities. Amongst the pool of naturally derived phytochemicals, a linear link between calculated descriptors and anti-dengue virus efficacy was found by QSAR analysis. Descriptors include hydrogen bond donors, acceptors, log *P* value, number of bonds, rotatable bonds, hydrophilic volume, totally positive and negative partial charge, SlogP, etc. The authors selected the following flavonoids showing the best docking score: quercetin 3-*O*-(2″,3″-digalloyl)-*β*-d-galactopyranoside, quercetin 3-*O*-*α*-(6″-caffeoylglucosyl-*β*-1,2-rhamnoside), schaftoside, myricetin, quercetin 3-sulfate, eriocitrin, catiguanin B, 4′,5,7-trihydroxy-3-methoxyflavone-7-*Oα*-l-arabinofuranosyl(1 → 6)-*β*-d-glucopyranoside, wogonin 7-*O*-*β*-d-glucuronide, and silychristin, for the QSAR study. QSAR analysis revealed that logP values have a positive correlation with biological activity. Based on the obtained results, a common pharmacophoric scaffold was proposed, as follows (Figure 5).

To date, numerous natural product-derived phytochemicals have been explored for anti-dengue activities. Amongst them, quercetin was the first well-studied natural chromene derivative against the DENV. It is primarily distributed in plants, abundantly in fruits, leaves, roots, barks, and flowers. Quercetin is a polyhydroxy flavonoid that has been reported for its effectiveness against influenza virus [62] and for herpes simplex virus and morin against equine herpes virus [63]. Based on these promising potential results, many reports are available, wherein quercetin is used as a positive control in the screening of anti-dengue effects.

**Figure 6:**
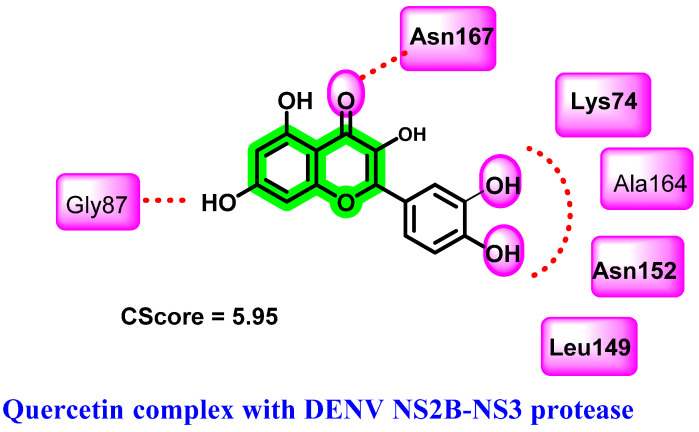
Hydrogen bonding interactions of quercetin with DENV NS2B-NS3 protease.

Based on the platelet count increment associated with the administration of *Carica papaya* leaves, major phytochemicals belonging to chromene derivatives were isolated and their anti-DENV activity evaluated [64]. Molecular modeling studies suggested that quercetin is the key phytochemical for exerting a DENV inhibition, where a stable binding pattern is observed with NS2B-NS3 protease. Binding affinities were evaluated using the consensus scoring function (CScore) and it was highest for quercetin (5.95). A prominent hydrogen bonding interaction with Asn152, Ala164, Lys74, Asn167, Leu149, and Gly87 of NS2B-NS3 with quercetin was revealed (Figure 6). In silico ADMET prediction (Lipinski’s rule of five) further suggested the drug-likeness possibilities of quercetin. Further in vivo studies were conducted to determine the effects on platelet count. A significant rise in platelet count has been observed with the administration of leaf extract of *Carica papaya* (at 400 mg/Kg and 800 mg/Kg concentrations) to a cyclophosphamide-induced rat [60]. *Isatis tinctoria*, a traditional Chinese medicinal herb used to treat dengue virus infection, also contains quercetin as one of its active ingredients [40].

**Figure 7:**
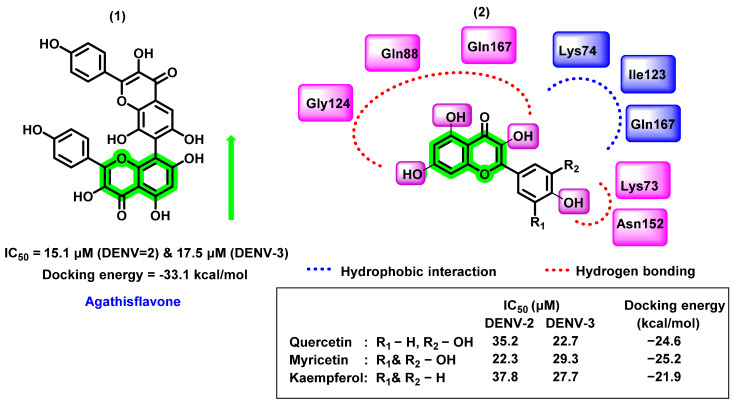
(**1**) Non-competitive NS2B-NS3 protease inhibitor (**2**) chromene derivatives complex with the allosteric binding site of NS2B-NS3 protease showing hydrogen bonding and hydrophobic interactions.

In another study, Sousa et al. [65] evaluated six different flavonoids (agathisflavone, quercitrin, isoquercitrin, myricetin, quercetin hydrate, and kaempferol) for their DENV inhibitory properties. Amongst the screened analogues, agathisflavone, a biflavonoid, was found to have potential for DENV-2 and DENV-3 strains (noncompetitive NS2B-NS3 serine protease inhibitor). The inhibitory activity of these molecules has been identified by using a substrate-free microscale thermophoresis (MST) assay. The IC_50_ value was 15.1 and 17.5 μM against DENV-2 and DENV-3 strains. By analyzing the IC_50_ values, not much difference was found between the glycosylated flavonols and the aglycones. In vitro inhibitory potential was further correlated with molecular modeling reports. NS2B-NS3 protein, derived from DENV-3 serotype with the co-crystallized ligand, Bz-nKRR-H (PDB ID:3U1I), was used as a protein target and molecules were docked to the specific allosteric binding point adjacent to the catalytic triad. The 3,5,7,4′- hydroxyl group formed hydrogen bonding with Gln88, Gln167, Gly124, Asn152, and Lys73 residues in the binding pocket. Hydrophobic interactions were also found with Lys74, Ile123, and Gln167. The meta hydroxyl group did not exhibit any interaction with the target protein (Figure 7). A reversible noncompetitive inhibitory nature of agathisflavone and myricetin was derived through the enzyme kinetic analysis (DENV-2 NS2B-NS3 protease). On the contrary, Powers and Setzer [66] reported that the agathisflavone has lower docking binding affinity (−33.1 kcal/mol) towards DENV protease, also a violation of Lipinski’s rule of five since its molecular weight is 522.46 g/mol.

**Figure 8:**
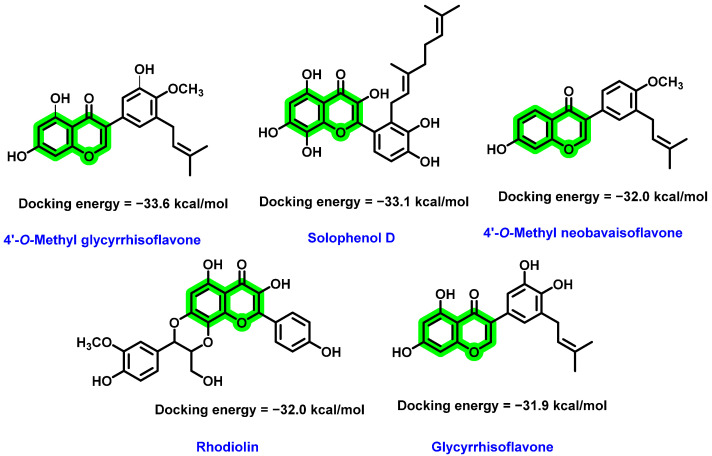
Docking of different flavonoids with DENV NS2B-NS3 protease with their docking score.

In 2016, Powers and Setzer [66] performed the virtual screening of 2194 phytochemicals (including 349 flavonoids and 120 isoflavonoids) against various DENV protein targets as follows: crystal or NMR structures of NS2B-NS3protease, NS3 helicase, envelope protein, NS5 RdRp, and methyltransferase (MTase). Amongst those screened analogues five chromene derivatives exhibited strong binding interactions with the catalytic triad of NS2B-NS3 protease target (PDB ID: 2M9P and 2M9Q) (Figure 8). None of the flavonoid ligands have notable binding affinities with the DENV helicase and RdRp. The docking results showed excellent binding of polyphenolic compounds with the DENV target proteins. It is interesting to note that the substitution of isoprene functionalities (prenyl and geranyl) resulted in an enhancement in binding potential towards the target.

A pool of flavonoids derived from *Azadirachta indica* was virtually screened for anti-DENV activity on the NS2B/NS3 protease triad (His51, Asp75, and Ser135) [67]. The crystal structure of NS2B-NS3 protease (ID: 2FOM) was selected from PDB. All the screened analogues interacted to the catalytic sites comparatively in a similar manner. When compared to the reference molecule quercetin (−6.94 kcal/mol), epicatechin (−7.622 kcal/mol), hyperoside (−7.879 kcal/mol), rutin (−9.324 kcal/mol), and kaempferol-3-*O*-rutinoside (−9.555 kcal/mol) possessed a higher binding potential.

**Figure 9:**
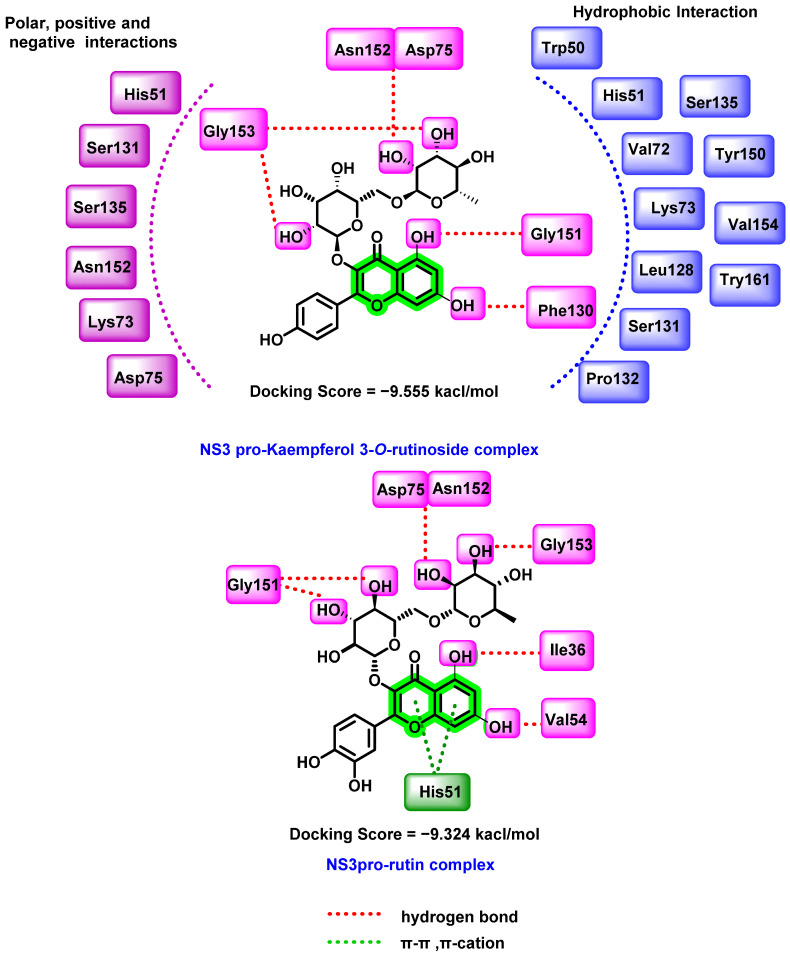
Docking interactions of kaempferol-3-*O*-rutinoside, rutin with the DENV NS3 protein.

The NS3 protein-kaempferol-3-*O*-rutinoside complex (Figure 9) displayed substantial hydrogen bonding and hydrophobic interactions and polar interactions. In molecular dynamic simulation studies, the RMSF, RMSD, and protein–ligand interaction map data were evaluated and found that the cα-carbon of NS3 protease interacted with the flavonoids. The nucleophilic attack of a hydroxyl group of Ser135 mediated by His51 is required to initiate the proteolytic activity of the NS3 proenzyme. Any hydrogen bonding with any residues in the catalytic triad disrupts the electron transport across the His51 imidazole nitrogen and Asp75 carboxyl group, resulting in inhibitory activity. A similar trend was observed with the NS3pro-rutin complex as well. In both of the most active analogues, sugar attachment resulted in interaction with the catalytic domain amino acids, which suggested the overall inhibitory potential of these analogues against NS3 protein.

Based on these promising results, the nucleophilic and electrophilic regions required for essential intermolecular interactions with the target (NS3 pro) were identified using the density functional theory (DFT). The results suggested that these phytochemicals were more stable and chemically inert than the quercetin. Free binding energy calculation followed by ADMET prediction further confirmed the potential of kaempferol-3-*O*-rutinoside as the lead candidate. An MTT assay was performed to check the cell viability of kaempferol 3-*O*-rutinoside and the reference compound (quercetin) against BHK-21 cells [67]. Kaempferol 3-*O*-rutinoside showed no significant cytotoxicity until 100 µM concentration. Quercetin was found to be 100% toxic at a concentration of 100 µM. Based on the cytotoxicity profile, a 10–100 µM concentration range for kaempferol 3-*O*-rutinoside was used for the antiviral potential evaluation. The antiviral performance was assessed against the DENV-2 strain and detected the intracellular virus via the immunofluorescence imaging technique. The treatment with flavonoids inhibited DENV-2 infectivity in a dose-dependent manner. Kaempferol-3-*O*-rutinoside was found to be more highly effective (77.0% at 100 µM) than the reference compound quercetin (60.6% at 100 µM).

In another study, the antiviral assay test for the aqueous extract (at a concentration of 10–40 µg/mL) of the *Houttuynia cordata* plant was established for DENV-2 inhibition [68], which contains hyperoside as the major component. The extract showed an EC_50_ = 0.8 mg/mL, very low cytotoxicity CC_50_ = 1.24 mg/mL in LLC-MK2 cells, and very high SI = 1550.36 [69]. Inhibitory activity of the aqueous extract of *Houttuynia cordata* on DENV-2 was attributable to direct destruction of virus particles before cell infection as well as viral entry into the cells after adsorption. The anti-dengue virus activity of hyperoside could be linked to both hydroxyl groups and glycosidic (galactoside) side groups. It is speculated that the glycosides present in the hyperoside might interact with various viral components.

### 8.3. Chromene Analogues Acting on NS5 Protein

Numerous reports have explored the anti-dengue properties of various chromene analogues that act through NS5 protein.

**Figure 10:**
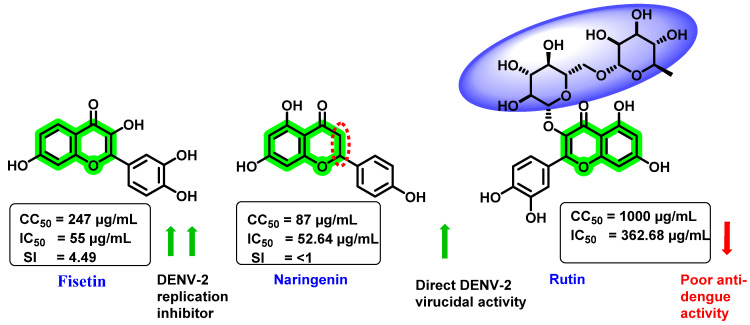
Structure, CC_50,_ and IC_50_ of fisetin, naringenin, and rutin.

In vitro activity of three natural flavonoids (fisetin, naringenin, and rutin) against DENV was investigated (Figure 10) by Zandi et al. [70]. Foci-forming unit-reduction assay (FFURA) and quantitative reverse transcriptase-polymerase chain reaction (qRT-PCR) were used to assess the in vitro effectivity. Amongst these analogues, fisetin exhibited a dose-dependent inhibitory activity in DENV replication, while naringenin showed direct DENV-2 virucidal activity (IC_50_ = 52.64 µg/mL). Further, rutin exerted poor inhibitory activity. The CC_50_ value against Vero cells was 247, 87, and >1000 µg/mL, respectively, for fisetin, naringenin, and rutin. After virus adsorption to the Vero cells, fisetin exhibited an IC_50_ value of 55 µg/mL with SI of 4.49. However, fisetin treatment (5 h prior to viral infection) demonstrated an IC_50_ value and SI of 43.12 µg/mL and 5.72. At a 50 µg/mL concentration, fisetin decreased the DENV-2 RNA level by 65%, while naringenin reduced it by 50%. The actual mechanism for the inhibitory potential of fisetin is uncertain. However, it is suggested that forming a complex with the viral genome prevents further replication or interferes with the RdRp of the NS5 protein. Naringenin has high cytotoxicity (87 µg/mL) and SI < 1. The inactivity of naringenin may be due to unsaturation at the C2–C3 bond, whereas that of rutin may be due to glycoside group attachment at the C3 position. However, contradictory results were found in in silico [67] and in vitro [71] studies, which showed rutin as a potent DENV NS3 pro inhibitor with an IC_50_ of 362.68 μg/mL.

Molecular modeling studies suggested that the polyphenolic groups present in quercetin and fisetin favor the anti-dengue virus activity. These compounds have interactions with NS5 RdRp and inhibited various stages of viral replication [72,73]. Further attachment of sugar moieties resulted in detrimental effects towards DENV.

Another critical study related to flavonoids was based on biflavonoids isolated from natural sources that showed potent inhibition against DENV-NS5 RdRp [74,75]. Coulerie P et al. evaluated the structure–activity relationship of 23 compounds, including four biflavonoids isolated from *Dacrydium balansae* (amentoflavone, podocarpusflavone A, isoginkgetin, and hinokiflavone), six biflavonoids isolated from *Dacrydium araucarioides* (robustaflavone, sotetsuflavone, etc.), ten apigenin derivatives, hexamethyl-amentoflavone, and cupressuflavone.

**Figure 11:**
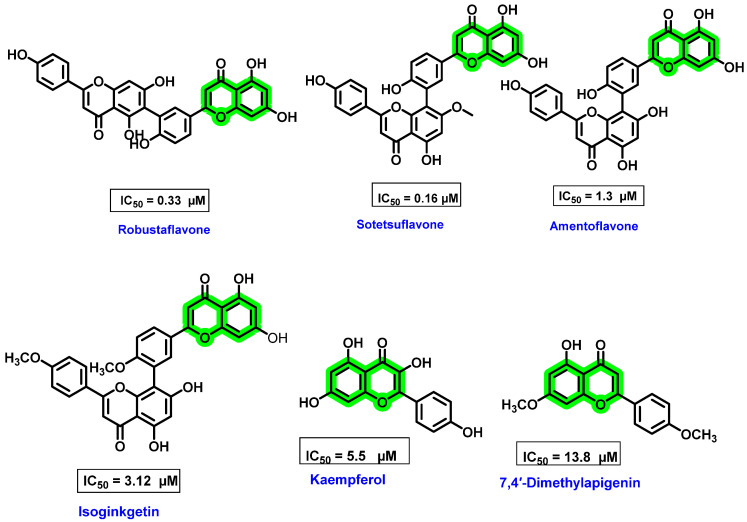
Structure of biflavonoids isolated from *Dacrydium* sp. and flavonoid monomers showing inhibitory activity against DENV NS5 RdRp and their IC_50_ values.

The nucleotide incorporation assay method was used for in vitro analysis, wherein radio-labeled guanosine was incorporated into a homopolymeric cytosine RNA template. 3′dGTP was used as a negative control and ribavirin as a positive control [74]. The in vitro assay showed that monomethylated amentoflavone exerted a lower IC_50_ value than amentoflavone and its di/tri-methylated derivatives, apigenin and its methylated derivatives. Methylation at the 7,7″ position showed maximum inhibitory activity [75]. Modifications in the position and number of methoxy groups caused a change in polarity and, thus, in turn, affects the hydrogen bond formation ability with NS5 RdRp. Among the substituted apigenin derivatives, 7,4″-dimethylapigenin has better inhibitory activity against polymerase (at a concentration of 50 μΜ 92% inhibition). The increase in the inhibitory activity is mainly due to oxygenated substituents on the A, B, and C rings of flavones. The anti-dengue virus potential is significantly affected by the position and number of methylations on the biflavonoid skeleton. Robustaflavone (IC_50_ = 0.33μΜ) and sotetsuflavone (7″-*O*-methylamentoflavone, IC_50_ = 0.16 μΜ) are the two highly effective biflavonoid polymerase inhibitors (Figure 11). Hinokiflavone was also a potent DENV inhibitor, but it was highly cytotoxic in nature. All these results highlighted the potential of biflavonoid analogues for exhibiting anti-dengue activities.

**Figure 12:**
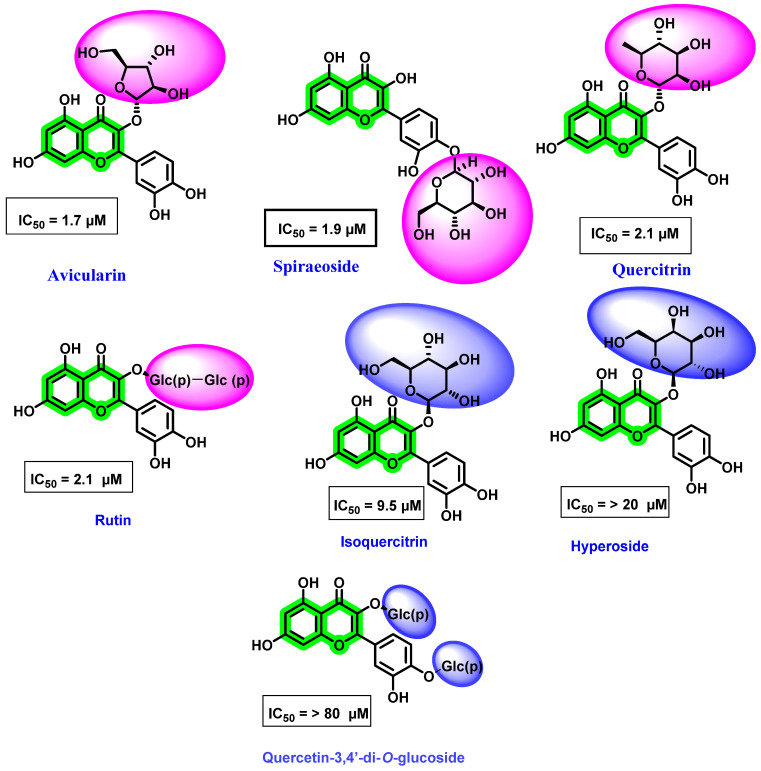
Structure of different flavonoids isolated from *Carpolepis laurifolia* leaf and commercially procured flavonoid derivatives active against DENV NS5 RdRp and their IC_50_ values. Pink indicates the most active position of glycoside attachment and blue indicates the position of glycoside with weaker inhibition.

Antoine et al. [76] evaluated various natural flavonoids against the DENV NS5 RdRp. Amongst the screened flavonoids, five were obtained from *Carpolepis laurifolia* leaf extract (quercetin, 6-methylapigenin-7-methylether, avicularin, quercitrin, and hyperoside), while four were commercially procured (isoquercitrin, spiraeoside, quercetin-3,4′-di-*O*-glucoside, and rutin) (Figure 12). It is noteworthy that all these flavonoids possessed a sugar unit attachment at various positions. Amongst the screened analogues, avicularin (with rhamnose sugar) and quercitrin (with arabinose sugar) demonstrated potential activity towards DENV RdRp, while hyperoside and isoquercitrin, with galactose and glucose at the third position of quercetin, respectively, showed poor inhibitory potentials. Furthermore, spiraeoside with glucose at the C-4′ place displayed inhibitory activity, while quercetin-3, 4′-di-*O*-glucoside with another glucose substituent at the C-3 position possessed a lesser inhibitory effect. These results were in contrast with the reports of Zandi et al. [70], wherein sugar attachment possessed a weaker potential.

In another report, baicalein was isolated from the roots of the *Scutellaria baicalensis* by microwave-assisted extraction and its DENV inhibitory activity was evaluated [77]. It possessed inhibitory activity against four serotypes of DENV (DENV-1 to DENV-4). Results highlighted that this compound has the potential to interfere with all steps in the viral replication process, as well as the DENV-2 RNA polymerase [78,79]. It is probable that baicalein reduces DENV replication *viz.* interfering with DENV-2 RNA polymerase and/or binding to viral RNA. Before the viral adsorption, it showed a potential IC_50_ value of 6.46 μg/mL (SI = 17.8) in Vero cells. It was administered before 5 h and continued up to 4 days after viral infection with an IC_50_ value of 5.39 μg/mL (SI = 21.3). It had an IC_50_ of 1.55 μg/mL for direct DENV inhibitory action and an IC_50_ of 7.14 μg/mL for preventing adsorption activity. The binding affinity was found to be −8.1 kcal/mol.

Anusuya et al. [80] predicted the non-nucleoside inhibitory activity of quercetin 3-(6″-(E)-*p*-coumaroylsophoroside)-7-rhamnoside on the dengue virus RNA-dependent RNA polymerase target (PDB ID: 3VWS) and validated the results using molecular dynamics simulation studies. The polymerase must alter its conformation from closed to open before the replication process. Non-nucleoside inhibitors retain the polymerase in closed confirmation all of the time, which results in the inhibition of DENV. Their designing strategy mainly involves the attachment of various electron-donating and electron-withdrawing groups at specific positions in the quercetin derivatives. The designed 185 analogues were studied for their binding affinity towards DENV NS5 RdRp. Quercetin showed a docking score of −7.84 kcal/mol, while attachment of –CHO substituent at C_5′_ of the B ring (15EW9) resulted in an enhancement in the docking score (−8.37 kcal/mol). Numerous interactions, such as hydrogen bonding, hydrophobic and π–cation interaction, and coulomb energy, were revealed in the docking analysis. The amino acid residues in the polymerase protein essential for binding are Ser 600 (identifies the nucleotide substrate) and Thr413 (controlling the ssRNA template). The derivatives of quercetin interfered with this and inhibited the polymerase function. The binding free energy of 15EW9 (−54.86 kcal/mol) was lower than quercetin (−41.24 kcal/mol) in a comprehensive evaluation. The coulomb energy related to electrostatic interaction was the reason for this predominant difference in the binding free energy.

**Figure 13:**
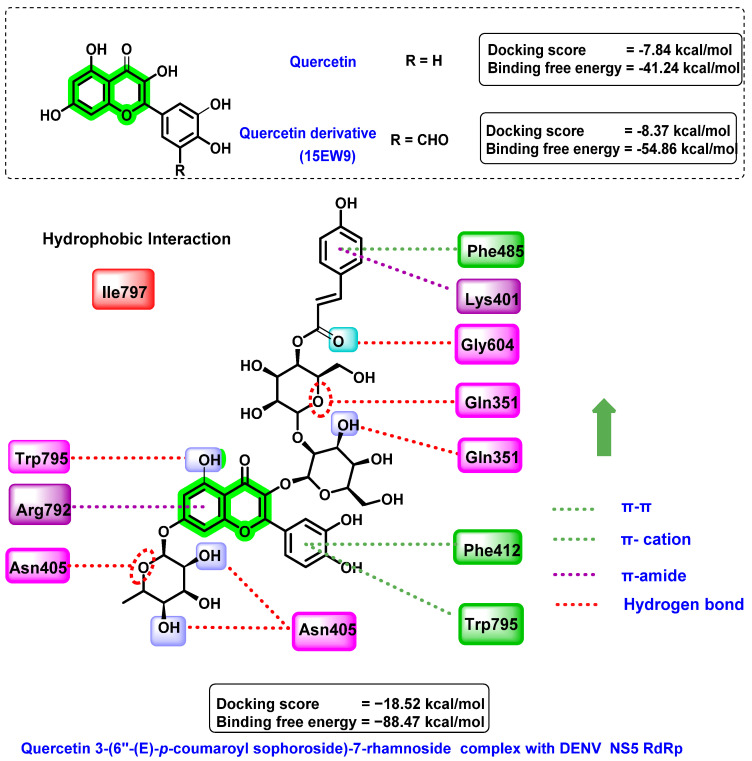
Docking interactions of quercetin and its derivatives with DENV NS5 RdRp.

Based on these promising results, a further set of similar quercetin structures was retrieved from the PubChem database and screened for the identification of anti-DENV lead compounds. Quercetin 3-(6″-(E)-*p*-coumaroyl-sophoroside)-7-rhamnoside has a higher docking affinity (−18.52 kcal/mol) than quercetin and its derivatives (Figure 13). Various interactions, such as hydrogen bonding, hydrophobic interactions, and π–cation interactions, were formed by the screened analogue towards the targets. Interactions with amino acid residues, such as Trp795, Arg792, and Gln351, were found to be critical for their anti-dengue virus activity. Trp795 performs a vital role in the entry and release of RNA template as well as stabilization of the polymerase–template complex. Quercetin 3-(6″-(E)-*p*-coumaroyl-sophoroside)-7-rhamnoside has a free binding energy of −88.47 kcal/mol. From the molecular dynamic simulation studies, it was found that water bridges and hydrogen bonds were essential for the stability of the polymerase–ligand complex. Further pharmacokinetic studies suggested the druggability of these molecules against dengue fever.

### 8.4. Chromene Analogues Acting on Multiple Targets

#### 8.4.1. Chromene Analogues Acting on DENV-2 My/DENV-E Protein

In vitro analysis of ethyl acetate extract of aerial parts of *Houttuynia cordata,* a plant native to eastern Asia, and its subsequent studies resulted in the identification of three flavonoids (quercetin, quercitrin, and rutin) as potential inhibitors against DENV-2. Previous reports were available for the promising role of these plant materials against DENV, wherein steam distillate of *H*. *cordata* can subdue an enveloped virus but cannot disrupt a non-encapsulated virus [39]. Based on these observations, the ethyl acetate fraction is screened against the enveloped DENV viruses. Plaque-reduction neutralization test, using BHK-21 (baby hamster kidney) fibroblasts cells, was used for biological evaluation [81]. The ethyl acetate extract showed an IC_50_ = 7.5 μg/mL, SI > 22.22 with no cytotoxicity, while the isolated quercetin exhibited an IC_50_ = 176.76 μg/mL, SI = 0.88, and CC_50_ = 155.38 μg/mL. Quercetrin had little impact on DENV-2, whereas rutin had no effect. The combination of quercetin and quercetrin showed an enhanced inhibitory effect on DENV-2 with an IC_50_ = 158.21 μg/mL, SI = 1.71, and CC_50_ = 270 μg/mL. In vivo studies were also conducted for acute oral toxicity study in mice for ethyl acetate fraction. With a maximum oral dose of 2000 μg/mL, there was no evidence of acute toxicity and no variation in histopathological examinations between the control group and the testing group.

**Figure 14:**
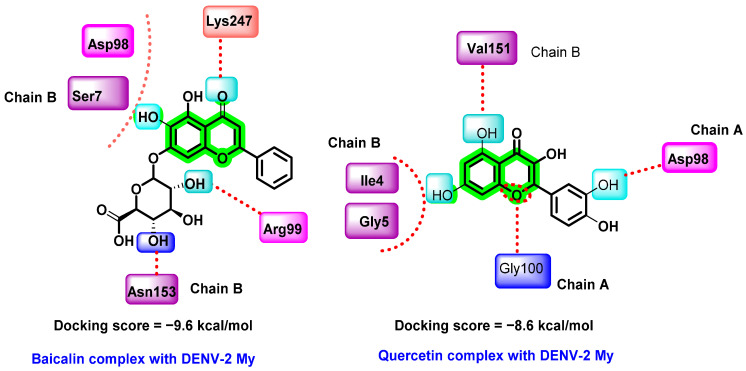
Hydrogen bonding interactions of baicalin and quercetin complex with DENV-2 My.

In another report, in silico binding affinities of various flavonoids with DENV-2 E protein were studied by Ismail et al. The selected flavonoids include baicalein, baicalin, epigallocatechin gallate (EGCG), fisetin, flavone, glabranine, hyperoside, ladanein, and quercetin (Figure 14) [72]. The structure of the E protein was prepared by homology modeling, isolated from DENV-2 Malaysia (DENV-2 My). The flavonoid consensus binding pocket was generated by the residues 4–9 and 151–154 from domain I of chain B and residues 98–103 and 244–247 from domain II of chain A.

Among those screened phytochemicals, baicalin exhibited the highest binding affinity (−9.6 kcal/mol) with the E protein, followed by quercetin (−8.6 kcal/mol) and baicalein (−8.1 kcal/mol). Baicalin is a flavonoid isolated from a Chinese herbal medicine, *Scutellaria baicalensis.* It is also a metabolic product of baicalein isolated from the plant’s roots. Previous reports on the virucidal and anti-adsorption properties of baicalin against DENV-2 (NGC strain) are available [82]. In this study, MTT assay was used for cytotoxicity analysis and a foci FFURA test was used to determine in vitro antiviral efficacy, which was further confirmed with the help of qRT-PCR [44,78,82]. Baicalin inhibited the DENV-2 binding to the Vero cells at an IC_50_ of 18.07 μg/mL, suppressed viral replication at 13.50 μg/mL, and had a virucidal effect against DENV-2 extracellular particles at 8.74 μg/mL. The virucidal activity of baicalin and baicalein lowers the circulating DENV particles during the viremic phase of dengue. The docking result showed quercetin docked to the consensus binding pocket of DENV-2 E protein with a binding affinity of −8.6 kcal/mol.

#### 8.4.2. Chromene Analogues Acting on DENV Polymerase and DENV Protease

In another study, Anusuya et al. performed a quantitative structure–activity relationship (QSAR) of quercetin analogues (flavones, flavonols, flavanones, and biflavonoids) using multiple linear regression (MLR) models. Three molecular descriptors, such as hydrogen bond acceptor, branching index concerning size, and the electro topological state descriptor related to atom type carbon, were used for the construction of MLR [83]. The MLR created a linear equation that connects the biological activity (dependent variable) to the chosen descriptors (independent variables) and fits all of the data points in the scatter diagram.

pIC_50_ = 0.115 nwHBa + 215.975 ETA_EtaP_B_RC + 1.68 Saaac − 2.988

n = 32, R = 0.906, R^2^ = 0.821, Adjusted R^2^ = 0.802, SE = 0.2989

n = number of flavonoids in the dataset, R = Pearson correlation coefficient, R^2^ = coefficient of determination, adjusted R^2^ = modified version of R^2^ for the number of descriptors in the model, and SE = standard error.

This study revealed that the hydrogen bond acceptor covers nearly half of the variance in the dependent characteristic, whereas the branching index with regard to size and the electro topological state descriptor connected to atom-type carbon describe the remaining 35% and 15% of the variance, respectively. Two hydrogen bond acceptors in active flavonoids are the C1-oxygen and C4-carbonyl groups, which form interaction with Gln350, Gln351, and Arg352 or Val411, Phe412, and Thr413 residues of polymerase (PDB ID: 3VWS). Among the selected set, all biflavonoids showed activity due to the descriptor branching index with size. However, the position of dimerization distinguishes biflavonoids in terms of potency, making them more effective when dimerized at 3′–8″, 3′–6″, and 4′–6″ than when dimerized at 8–8″. The electrotopological state descriptor for atom carbon considers the electronegativity difference between the carbon atom and the adjacent atoms, which are connected by bonds. It may also vary due to branching, cyclicity, and changes in the relative position of different groups.

**Figure 15:**
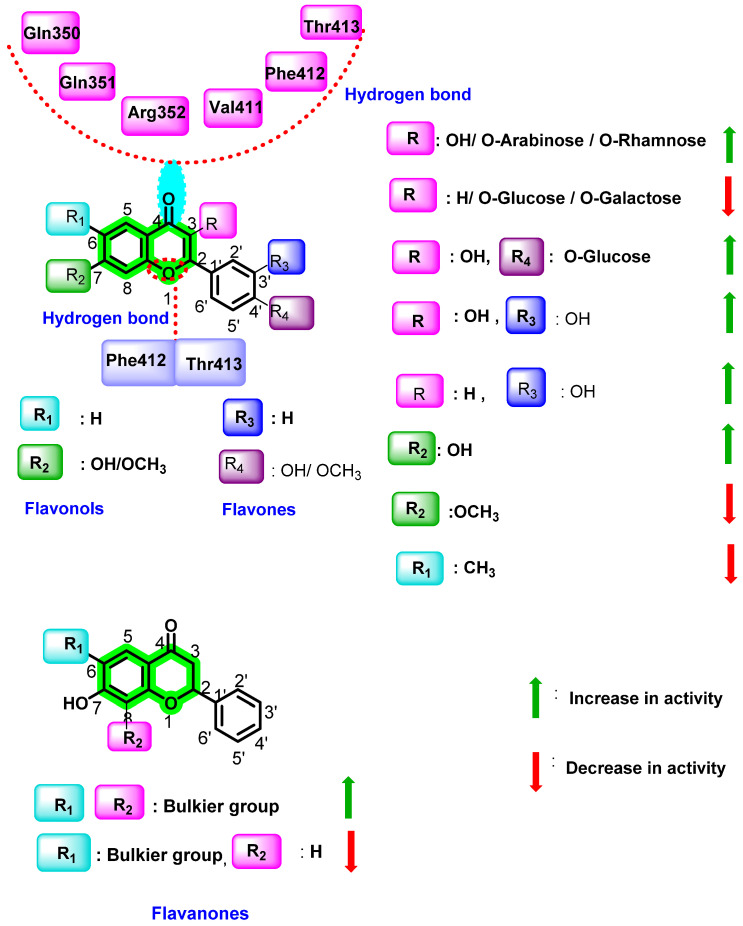
QSAR of flavonols, flavanones, and flavones.

By testing 32 different flavonoids (flavonol, flavones, flavanone, and biflavonoid) with IC_50_ values ranging from 0.16 to 80 nM, it was revealed that dimerization or the addition of a glycosyl group or a long side-chain in chromene improved the anti-dengue virus effect of flavonoids [83]. Further, 5-hydroxy-3,3′,4′,6,7,8-hexamethylflavone (IC_50_ = 4.9 nM), 4′,5,6,7-tetramethylflavone (IC_50_ = 7.7 nM), and 7″-methylamentoflavone (IC_50_ = 23.4 nM) were found to be the potential inhibitors of DENV polymerase. In flavones, -OH group at position 7 (IC_50_ = 5.56 nM) increases antiviral activity, whereas the bulky -OCH_3_ group (IC_50_ = 5.88 nM) decreases activity and -CH_3_ at position 6 did not possess any effect. The -OH group at 3 and 3′ positions improves the antiviral activity in flavonols. The detailed interaction behavior as well as the inhibition activity are represented in Figure 15.

#### 8.4.3. Chromene Analogues Acting on DENV E Protein and NS5

Trujillo-Correa et al. [84] identified anti-DENV phytochemicals from ethanolic extracts of the bark of the Columbian plant *Psidium gujava* using bioprospecting studies. The isolated phytochemicals include gallic acid, quercetin, catechin, naringin, and hesperidin. In vitro, DENV inhibition analysis has been screened by using pre- and post-treatment approaches. Except for hesperidin, the remaining compounds were found to be effective for DENV at a concentration below 100 μg/mL. Amongst these compounds, quercetin expressed the most selective (EC_50_ = 19.2 μg/mL, SI = 34.3) and active inhibition against DENV (100% inhibition), while 91.8, 67.3, and 64.5 % inhibition was exerted by catechin (EC_50_ = 33.7 μg/mL, SI = 24.8), gallic acid (EC_50_ = 25.8 μg/mL, SI = 25.1), and naringin (EC_50_ = 4 7.9 μg/mL, SI = 13.5), respectively. In cytotoxicity screening, these analogues revealed a safer profile, catechin displayed the least cytotoxicity CC_50_ = 833.3 μg/mL, followed by quercetin 659.8 μg/mL and naringin, showing 646.8 μg/mL.

Molecular docking studies of these phytochemicals were performed against DENV E protein domain 3 (PDB ID: 3UZV) and NS5 polymerase (PDB ID: 2J7U). Suramin was used as a standard drug that exerted a docking score of −7.9 kcal/mol towards DENV E protein, naringin, and hesperidin exerted a higher docking score (−8.0 and −8.2 kcal/mol, respectively) than standard drug. While docking with NS5 polymerase, except gallic acid (−5.3 kcal/mol), all the compounds showed a better profile; suramin has a docking score of −12 kcal/mol and hesperedin, naringin, quercetin, and catechin showed a docking score of −8.8, −8.4, −7.8, and −7.2 kcal/mol, respectively.

### 8.5. Chromene Analogues Acting on Furin Enzyme

**Figure 16:**
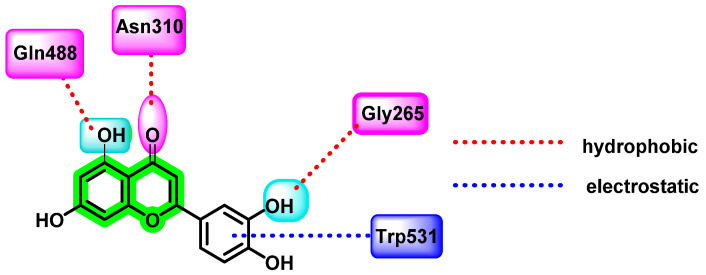
Luteolin complex with furin enzyme.

Another potent flavonoid, luteolin, the principal active component isolated from the plant *Viola yedoensis Makino*, has traditionally been used for fever treatment by Chinese physicians. It disrupts the viral maturation process by blocking the prM to M protein transition. Peng et al. investigated luteolin’s uncompetitive inhibitory action on the host proprotein convertase, furin enzyme, which is needed for viral maturation in the trans-Golgi network [85]. It also has a weak noncompetitive inhibitory activity on DENV NS2B-NS3 protease. Since furin is considered the best target for anti-dengue virus activity, the authors evaluated the cleavage of immature wild-type viruses and mutant viruses via the action of purified furin, with or without the addition of luteolin. For in vitro analysis, Huh-7 cells, BHK-21 cells, A549, HEK-293T cells, and Vero cell lines were used. Infectious analysis and Western blot analysis were also performed. In vivo antiviral studies were carried out, by administering 100 mg/Kg luteolin sample orally four times daily for four days to DENV-2-infected AG129 mice. The quantification of viral titer by qRT-PCR revealed a reduction in viremia as well as a reduction in the secretion of the cytokine interleukin 6. All four serotypes of DENV were inhibited by luteolin [86]. For in silico investigations, inhibitor-bound furin with PDB ID: 5MIM was used. Results indicated a hydrophobic connection between luteolin’s phenyl group and W531 (Trp) of the P-domain, and various electrostatic interactions between luteolin’s C3′ and G265 (Gly), C5 hydroxyl group and Q488 (Gln), and C4 carboxyl group and N310 (Asn) of the catalytic domain occurred (Figure 16).

### 8.6. Miscellaneous Reports

The antiviral potential of glycosylation or *O*-methylation on various -OH functional groups and substitution at the C2 position were explored by Rakers et al. [87] and Zandi et al. [70]. Results suggested that these factors play a significant role in enhancing antiviral activities. Glycosidic flavonoids are reported to possess antiviral activities. This characteristic behavior can be clearly demonstrated by considering quercetin.

**Figure 17:**
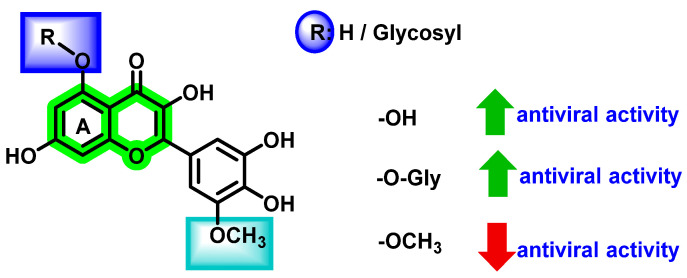
Effect of different substituent groups on flavonoid rings.

Although an increase in the number of hydroxyl groups does not vary the steric configuration, a change in the polarity and, thereby, its associated physicochemical properties, affected the overall antiviral activities. Proper steric configuration is essential for the alignment of analogues to the binding pocket while hydrogen donating functionalities are required for making hydrogen bonding interactions. Apart from that, incorporation of hydroxyl groups is responsible for the modification of solubility that, in turn, results in the in vivo absorption properties [88]. From the studies, it is evident that the presence of hydroxyl groups at the C_5_ and C_7_ positions of ring A increases the antiviral activity (Figure 17). It is speculated that these kinds of flavonoids exert their activities via various mechanisms, such as viral adsorption inhibition, entry of virus, virus attachment, RTase, integrase, protease, and DNA and RNA polymerases, which inhibit replication and protein complex formation.

**Figure 18:**
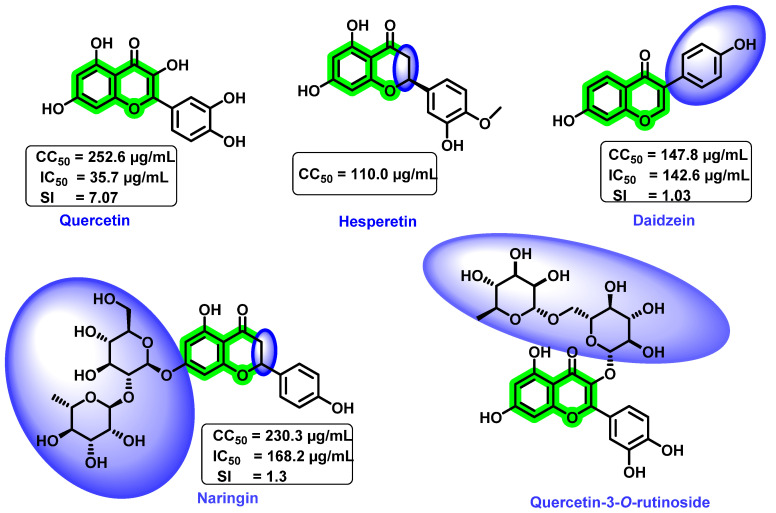
Examples of the effects of different substituent groups and their positions on the chromene nucleus. The blue highlighted area indicates the substituent group causes a reduction in anti-dengue virus activity.

Zandi et al. [89] determined the preventive, postadsorption, and direct virucidal effects of quercetin, naringin, hesperetin, and daidzein (Figure 18) against dengue (DENV-2). In vitro cytotoxicity study revealed that the quercetin and naringin exerted lower cytotoxicity towards Vero cells than the remaining screened analogues. The 50% cytotoxic concentration (CC_50_) value for quercetin, daidzein, naringin, and hesperetin was 252.6, 147.8, 230.3, and 110.0 µg/ML, respectively. Quercetin was more effective against replication of DENV-2 (IC_50_ = 35.7 µg/mL, selectivity index (SI) = 7.07) within the cell because it acts at the different processes of viral multiplication in the host cell. However, naringin (IC_50_ = 168.2 µg/mL, SI = 1.3) and hesperetin prevented the adsorption and attachment of dengue viruses, and these compounds were found to be inactive after the adsorption of DENV-2 to Vero cells. It is anticipated that the metabolism of the basic flavonoid might be the probable reason for the inactiveness after adsorption to Vero cells. In the case of daidzein (IC_50_ = 142.6 µg/mL, SI = 1.03), the attachment of the substituted aromatic ring on the basic scaffold plays a significant role in inhibitory activity. At a concentration of 50 µg/mL, quercetin decreased RNA production by 67% and daidzein reduced it by only 25.3%. Continuous treatment with quercetin from 5 h before virus infection to 4 days after infection resulted in an IC_50_ = 28.9 µg/mL and SI = 8.74; however, the remaining compounds had no anti-dengue virus activity.

Though an actual mechanism of quercetin for the inhibitory potential is yet to be revealed, it is speculated that the formation of a complex with RNA, thereby inhibiting the cellular RNA polymerase, might result in the inhibitory mechanism. Overall, the flavonol scaffold could inhibit DENV replication, whereas flavanone, its glycosides, and isoflavone did not affect viral replication. A further glycosylated derivative of quercetin, quercetin-3-*O*-rutinoside (Figure 18), demonstrated poor inhibitory activity [70]. From these results, it was clear that quercetin possessed DENV inhibitory potential. However, due to the lower bioavailability of quercetin, further development as a drug candidate is slowing down. Numerous other approaches for enhancing the overall bioavailability are being explored nowadays. They include combining with lipids and emulsifiers or the use of co-crystallization or esters of quercetin, etc. Moreover, the combination of quercetin with other potent anti-dengue virus agents was reported to possess synergistic inhibitory activity. In vitro analysis of a combination of α-glucosidase inhibitor, CM-10-18, with ribavirin (a standard antiviral medication) synergistically inhibited DENV infection [90].

**Figure 19:**
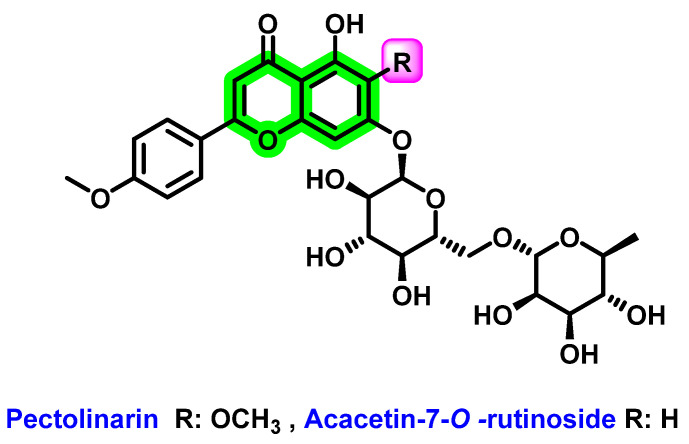
Structure of compounds extracted from *Distictella elongata* plant.

In vitro analysis of a mixture of pectolinarin and acacetin-7-*O*-rutinoside [91] (Figure 19), extracted from the fruits of *Distictella elongate,* showed eight-times more potent DENV-2 inhibitory activity (EC_50_ = 11.1 μg/ mL and SI > 45) than the isolated pectolinarin (EC_50_ = 86.4 μg/mL and SI = 4.6). The increase in activity was due to the contribution of acacetin-7-*O*-rutinoside. It was complicated to isolate this from the mixture. In pectolinarin, the drastic reduction in the anti-DENV activity may be because of -OCH_3_ substitution at the 6th position. For the cytotoxicity assay, LLCMK_2_ cells were used and the antiviral activity was determined by the MTT assay.

**Figure 20:**
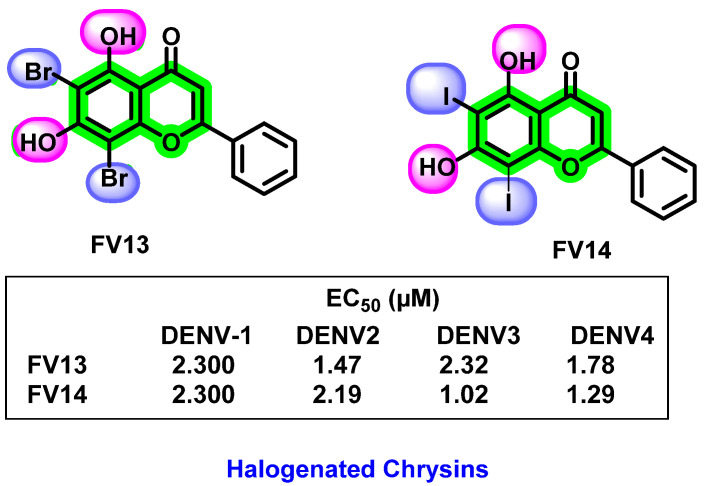
Structure and EC_50_ values of halogenated chrysins on four serotypes of DENV.

In 2017, the potent inhibitory activity of halogenated chrysins against DENV-1 to DENV-4 were reported [92]. 6,8-dibromo chrysin showed lower cytotoxicity than its corresponding iodinated chrysin. Based on studies on the multiple targets at the different processes of the viral life cycle, it was clear that the halogenated chrysin mainly affected the viral translation and replication stages. The potent biological activity against DENV may be due to the hydroxyl groups at the 5th and 7th positions and electronegative groups at the 6th and 8th positions of the flavones (Figure 20). In cell-based assays against DENV-2, halogenated chrysins are 20–100-times more efficient than the reported quercetin, fisetin, baicalein, baicalin, and naringenin. More than 80% of cells are viable by analyzing the cytotoxicity assay, assuming they are non-toxic and safer.

## 9. Conclusions

**Figure 21:**
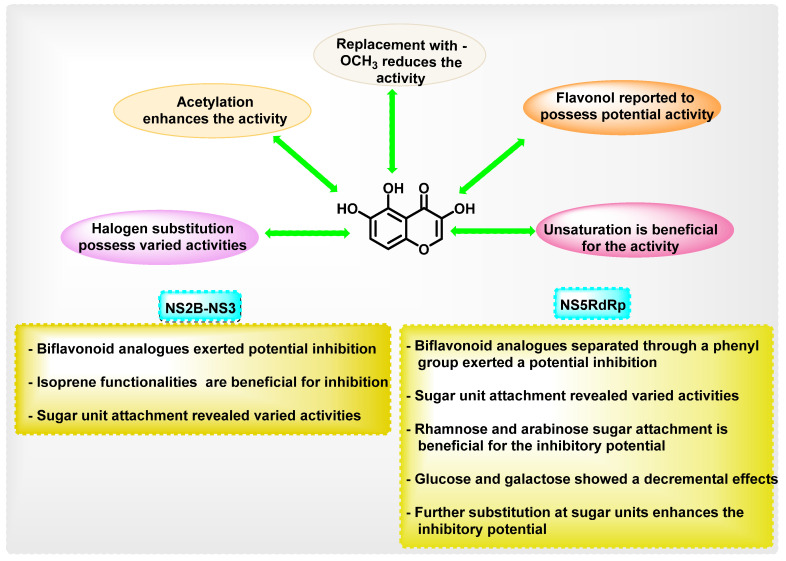
Summary of SAR points in dengue inhibition.

Chromene is a heterocyclic scaffold that is commonly employed in the development of new medicinal agents. Chromene derivatives have been reported to possess anti-cancer, antibacterial, antiviral, anti-inflammatory, anticholinesterase, monoamine oxidase (MAO) inhibition, etc. Many phytochemicals with the chromene nucleus have demonstrated antiviral properties. The overall structural activity relationships of various analogues are summarized in Figure 21.

The overall antiviral activity of the chromene scaffold is influenced by a variety of factors, such as the double bond (C2–C3) present in the C ring and the attachment of other essential pharmacophoric elements. The present review detailed the roles of different natural chromene scaffolds containing derivatives for their anti-dengue virus activities. Depending on the various chemical functionalities as well as their attachment positions, variation in the inhibitory potential is exerted. The C2–C3 double bond is essential for DENV inhibitory function; unsaturation at this bond in naringin and hesperidin showed a reduction in inhibitory potential. In quercetin and fisetin, the presence of the -OH group at the 3rd position enhanced the DENV inhibition, whereas, in luteolin, the absence of the 3-hydroxyl group showed a reduction in activity. When it is substituted with a glycosidic group at the 3rd position, an overall enhancement in the antiviral activity is seen in avicularin (rhamnose) and quircitrin (arabinose). However, the glucose and galactose substitutions showed a decrease in anti-dengue virus activity, as in isoquercitrin and hyperoside. In the case of rutin, a controversial result was obtained, wherein some in vitro research showed poor DENV inhibitory activity, whilst other reports showed an enhancement in anti-DENV potential (IC_50_ 2.1 μM, 362.68 μg/mL). In docking studies, all glycosidic attachments displayed a high docking score. In daidzein, exchanging the phenyl substituent from the second to the third position reduced inhibitory activity. Biflavonoids, such as agathisflavone, demonstrated high inhibitory potential against DENV. Amongst these reported phytochemicals, a few studies reported the in vivo efficacy, pharmacokinetics as well as pharmacodynamics efficacy; thus, a detailed exploration of these parameters is essential in the coming stages. Further, the attachment of sugar units was reported to possess a contrary result in many reports. Thus, a detailed study for exploring the potential of sugar units in anti-dengue properties needs to be performed. In addition to that, a multi-targeted approach that aims to focus on various dengue virus serotypes needs to be explored for the development of potential agents. Overall, further exploration of structural features in the chromene scaffold might result in potent anti-dengue virus agents, and this review will be helpful for medicinal chemists.

## Figures and Tables

**Table 1:** Structural features of DENV non-structural proteins and their respective functions.

Non-Structural Protein	Structural Features	Molecular Mass (KDa)	Functions
NS1	Multifunctional protein with the exposed hydrophobic region.	43–48	Role at the beginning stage of RNA replication, forming RNA replication complex [12,24] The secreted NS1 protein is used as a diagnostic biomarker since it is identified in the bloodstream on the first day of acute infection symptoms Most conserved protein, making it an ideal vaccine target [17,26]
NS2A	Hydrophobic protein. The N terminal is positioned inside the lumen of the ER, and the C terminal is in the cytoplasm [17]	42	In viral assembly by promoting viral RNA transport in vesicles Very few drug discovery studies have used DENV NS2A as an antiviral target [27,28] C terminal residues are involved in viral assembly and secretion, whereas N terminal residues are involved in the cytopathogenic activity [29]
NS2B	Hydrophobic protein Both N and C termini are present in the cytoplasm	15	Acts as a co-factor for NS3 proteolytic activity NS2B itself is not a direct target for antivirals, but binding with NS3 can be a good target for dengue inhibitors [30]
NS3	Multifunctional protein Acts as a serine protease and RNA helicase, adenosine triphosphatase (ATPase), and RNA triphosphatase (RTPase). It’s a trypsin-like serine protease with His51, Asp75, and Ser135 residues in the catalytic triad [30,31] 77% of the amino acids are identical in all types of DENV, so it acts as the best target for vaccine generation [11] Due to the planar nature, it is challenging to find potent inhibitors [32]	70	In RNA genome replication, assembly, and host-immune response invasion DENV NS2B/NS3 protease is an essential target for rational drug discovery Its union is vital for proper folding, protease functions, and immune suppression [33] The closed conformation state of the NS2B3in solution is needed for substrate identification and effective proteolysis
NS4A	Highly hydrophobic transmembrane ER protein. The N-terminal domain is present in the cytoplasm, while the C-terminal domain is present in the ER lumen [28] It has six helices, three of which are amphipathic and located in the N-terminal, and the remaining three form transmembrane helices and are present in the C-terminal region [34]	16	It is essential for viral multiplication to continue
NS4B	Hydrophobic protein The NS2B/NS3 serine protease is essential for generating NS4B during the C terminal cleavage and a cellular signalase for the N terminal cleavage of polyprotein precursors There is no detailed information about the crystal structure or the NMR structure of the NS4B protein [34]. In 2006, Miller et al. [35] generated a membrane topology model by biochemical analysis	27	The exact function of NS4B in viral replication is uncertain, and it is thought to be involved in innate host immunity and protein-protein interactions with other viral proteins.
NS5	The most conserved of the viral proteins The most potent enzymes for viral RNA production are RNA-dependent RNA polymerase (C-terminal region) and methyltransferase (N-terminal region) [17] Along with NS2B and NS3 protein, NS5 protein is found in an oligomeric state in the ER It can modify structural conformations without changing its active domains	103	It is essential for RNA genome replication Functions as a target for therapeutic and vaccine development

**Table 2:** Current status of drugs for dengue fever under clinical trials (source: ClinicalTrials.gov, accessed on 8 October 2022).

Drug	Sponsor	Trial Identifier	Phase	Location
AT-752	Atea Pharmaceuticals, Inc.	NCT05366439	I	New York, United States
AT-752 in dengue infected patients	Atea Pharmaceuticals, Inc.	NCT05466240	II	Brazil
AT-752 in healthy subjects	Atea Pharmaceuticals, Inc.	NCT04722627	I	Victoria, Australia
JNJ-64281802 (For Dengue prevention)	Janssen Research & Development, LLC	NCT05201794	II	Brazil
JNJ-64281802 (With confirmed dengue fever patients)	Janssen Research & Development, LLC	NCT04906980	II	Singapore
JNJ-64281802 against DENV-3	NIAID	NCT05048875	II	Maryland and Vermont, United States
Zanamivir–Prevent Vascular Permeability in Dengue (ZAP-DENGUE)	George Washington University	NCT04597437	Early Phase I	-
Ivermectin	Mahidol University	NCT02045069	II/III	-
Ivermectin in paediatric dengue patients	Mahidol University	NCT03432442	II	Bangkok, Thailand
Chloroquine	University of Sao Paulo	NCT00849602	I/II	Ribeirão Preto, SP, Brazil
Celgosivir	Singapore General Hospita	NCT01619969	I/II	Singapore
Melatonin	Ilocos Training and Regional Medical Centre	NCT05034809	II	-
Balapiravir	Hoffmann-La Roche	NCT01096576	I	Ho Chi Minh City, Vietnam
NT-proBNP and Troponin in Dengue children	Le PhoucTruyen	NCT04837430	-	Ho Chi Minh City, Vietnam
Montelukast	Phramongkutklao College of Medicine and Hospital	NCT04673422	II/III	Bangkok and Songkhla, Thailand
Ketotifen	National University Hospital, Singapore	NCT02673840	IV	Singapore
AV-1	AbViro LLC	NCT04273217	I	Texas, United States

## Data Availability

Not applicable.
